# A practical method for measuring planar spatial resolution with scatter on a CZT gamma camera

**DOI:** 10.1186/s40658-024-00656-w

**Published:** 2024-07-01

**Authors:** Corinne Barrau, Perrine Tylski

**Affiliations:** 1 Unité de Physique Médicale - secteur Médecine Nucléaire, CHU Nîmes, bât ICG, Place du Professeur Robert Debré, Nîmes Cedex 9 30029 France; 2 Service de Physique Médicale et Radioprotection, Groupement Hospitalier Est, Hospices Civils de Lyon, Bâtiment Pinel, 52 Bd Pinel, Lyon, Bron 69677 France

**Keywords:** Spatial resolution, CZT camera, Performance measurement

## Abstract

**Purpose:**

This study proposes a practical method for evaluating 2D spatial resolution with scatter on a CZT planar detector gamma camera, which is simpler and faster than the NEMA method. It is used to characterize the influence of distance on spatial resolution FWHM on a CZT camera equipped with a WEHR collimator.

**Methods:**

The practical method uses linear sources tilted with respect to the detector axes. The spatial resolution full width at half maximum (FWHM) with four tilt angles was compared to the FWHM evaluated using the NEMA NU1-2018 method. Spatial resolution FWHM was also assessed with tilted sources acquired at distances of 0 to 20 cm using a single angle, with and without the post-processing image enhancement proposed by the manufacturer.

**Results:**

Estimated spatial resolution FWHM with tilted sources was close to the spatial resolution FWHM estimated at 7.63 mm by the NEMA method, with deviations ranging from − 5.62 to 4.59% at 10 cm depending on the angle considered. The study of spatial resolution FWHM dependence on distance indicates that, for distances less than 3 cm, the FWHM no longer decreases with distance. The manufacturer’s post-processing reduces the FWHM by an average of 15%.

**Conclusion:**

The practical method is quicker to implement and gives comparable results to the NEMA reference method for spatial resolution FWHM. Evaluation of spatial resolution with linear sources at short distances from the collimator is limited by the collimator effect and signal digitization. The tilted source method can be used to measure spatial resolution quickly and easily under clinical conditions for CZT planar cameras.

## Introduction

Gamma cameras based on cadmium-zinc-telluride (CZT) technology have been used in clinical routine in nuclear medicine departments for about fifteen years now. CZT cameras were initially dedicated to cardiac explorations and commercialized in single photon emission computed tomography (SPECT), but are now capable of performing whole-body (WB) SPECT acquisitions [1]. Among these systems, a WB SPECT camera with planar CZT detectors, the Discovery NM/CT 670-CZT (2016) or 870-CZT (2019) system, is marketed by the company GEHC (General Electric Healthcare, Haïfa, Israel). This system is equipped with two pixelated planar detectors (130 CZT units of 2.46 mm-sized pixels arranged in a 51 cm × 39 cm field of view) and by default, a wide energy high resolution (WEHR) collimator with pixel geometry matched to collimator holes [2].

One of the most relevant indicators of planar image quality routinely used is extrinsic spatial resolution, which depends on collimator features, the distance from the source to its surface, and the intrinsic resolution of the detector. According to the NU1-2018 standard of the National Electrical Manufacturers Association (NEMA) [3], measuring planar spatial resolution with a gamma camera detector with a collimator relies on determining the full width at half maximum (FWHM) of a 30 mm profile perpendicular to a pseudo-linear source at 10 cm from the collimator. For CZT systems, due to the sampling effect of the pixelated detector, the signal measured depends strongly on the exact position of the line source with respect to the collimator hole. For this reason, the NEMA NU1-2012 and NEMA NU1-2018 standards require taking a set of measurements by moving the linear source tube in 1 mm stages over a distance of at least 10 mm and over the width of at least twice the detector pixel pitch for CZT systems. Several teams have followed this method of measuring and determining planar spatial resolution on pixelated detectors [4–6]. One team has published results at 10 cm on the GEHC NM/CT CZT 870 camera with a method close to NEMA, using a slightly different phantom [2].

However, to properly characterize the spatial resolution in different clinical configurations, spatial resolution should be measured with the collimator in scattering media at several distances from the detector. For this type of characterization, the NEMA reference method is time-consuming and difficult to implement.

We validated a practical, simple way of measuring, based on a tilted source line image, as proposed by Wieczorek [7]. The results obtained are compared and discussed with those obtained using the NEMA 2018 method and, additionally, at several source-collimator distances in the presence of scattering media. The impact of a post-processing image-enhancement solution, Clarity 2D, is proposed by the manufacturer [8]. Clarity 2D is a post-processing image enhancement system for planar images. After noise reduction and contrast enhancement, each processed pixel content is weighted from its native value by a user-selected factor expressed as a percentage. The study also focuses on the assessment of spatial resolution at close range, where the effect of digital sampling coupled with the collimator effect causes image artifacts, making it more difficult to evaluate the spatial resolution.

## Material and method

### Material

The NEMA NU1-2018 standard requires two capillary tubes with an inside diameter of ≤ 1 mm and an active filled length of at least 120 mm. We chose to take advantage of the camera’s large field of view to acquire more than two linear sources.

The line sources used were either capillary tubes, as recommended by the NEMA NU1-2018 standard or a fillable extension tube with an inside diameter of 1 mm, as recommended by the French Society of Medical Physics [9]. The activity concentration of Tc-99 m was 1 GBq/mL. For the fillable extension tube, we used a test object endorsed by the French Society of Medical Physics [9], consisting of a 1 cm thick PMMA slab with parallel slots, spaced at variable distances between 5 and 60 mm (PTW, Freiburg). Given the geometry of the test object, the spatial resolution was assessed in one direction at a time. Square PMMA (poly methyl methacrylate) blocks with 30 cm sides and 1–5 cm thickness were used as scattering media.

The acquisitions were performed on a GEHC NM/CT 670 CZT equipped with a WEHR collimator (hole length: 45 mm, septal thickness 0.2 mm, matched to a matrix of CZT pixels of 2.46 mm). The NU1-2018 NEMA standard requires spatial sampling to be less than 0.2 FWHM. We expected a spatial resolution of around 7.46 mm at 10 cm (as specified on the D670 CZT datasheet), which corresponds to a spatial sampling ≤ 1.49 mm. We chose to measure with a 512 × 512 matrix size, corresponding to a 1.1049 × 1.1049 mm² pixel size. The default interpolation method of the NM/CT 670 CZT system uses a Lanczos kernel.

### Measuring planar spatial resolution with scatter according to the NEMA protocol

**Fig. 1:**
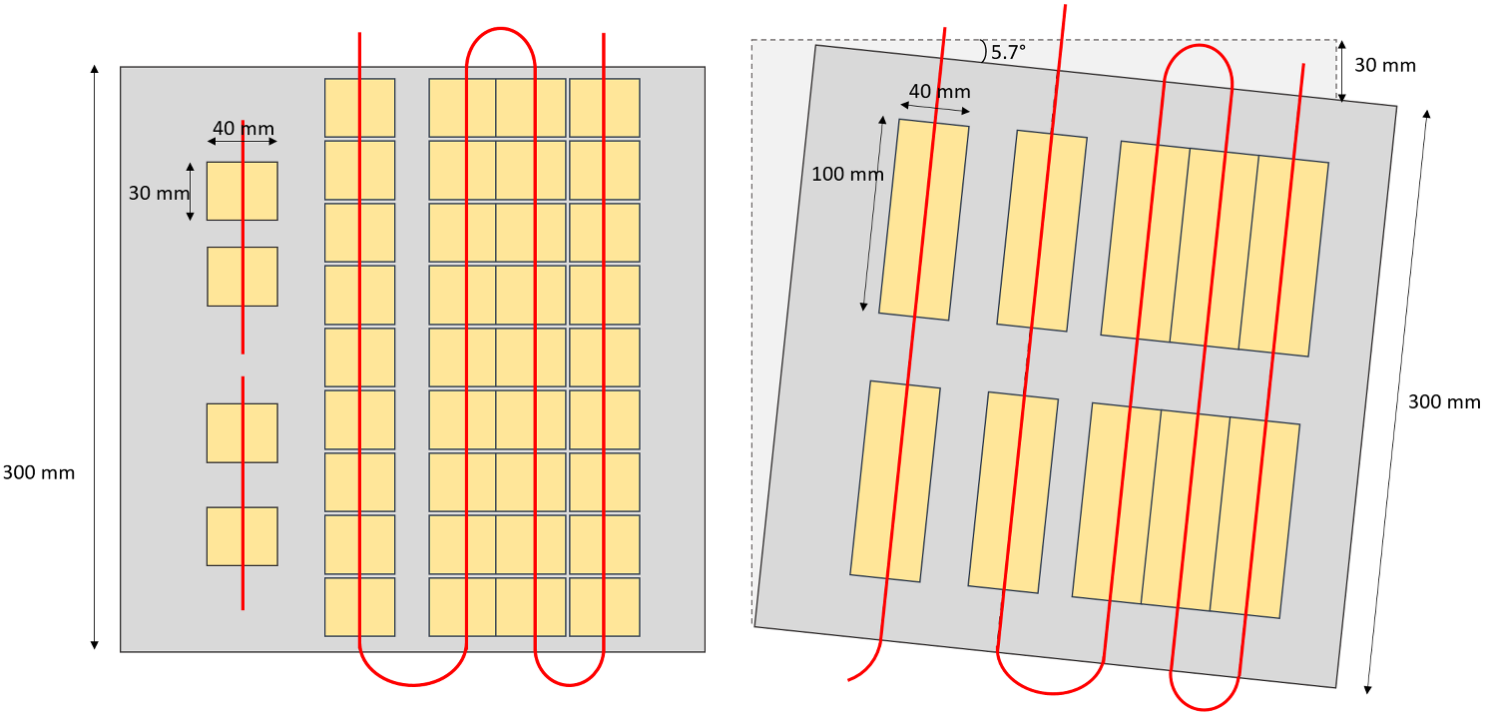
Schematic representation of linear sources for the NEMA (left) and tilted source (right) configurations. The tilted sources configuration is shown with a 30-mm shift corresponding to an angle of 5.7°. Given the convention of the axes of the figure, this configuration allows the evaluation of the spatial resolution in the x direction. The sources are represented in red and the 300 × 300 mm slab containing the source is light grey. The location and dimensions of the profile’s ROIs used for the NEMA configuration (40 ROIs of 40 × 30 mm²) and the tilted sources (1-pixel width profile and the ten 100-mm length ROIs used to average the FWHM) are represented in yellow

The sources were arranged parallel within the slotted slab described in the previous paragraph, with spacings between the sources of 60, 55, 35, and 35 mm, as represented in Fig. 1 (left).

The PMMA slab containing the sources was placed over a 10 cm PMMA block on the camera bed. A 10 cm PMMA scattering medium was placed over the sources according to the NEMA standard for resolution measurement.

The NEMA publication requires a set of measurements taken by moving the linear sources tube in 1 mm stages over a distance of at least 10 mm. This was achieved by carefully aligning the slab containing the sources with the detector and using a sheet of graph paper with a 1 mm grid fixed on the 10 cm PMMA block below.

Acquisition conditions were established to ensure that the maximum pixel value was at least 350 counts, in order to meet the NEMA criterion of a pixel value of at least 10,000 counts on a 30 mm summed profile. The energy window was set at 140.5 keV +/-7.5%. No post-processing was applied to the images.

The NEMA processing method was implemented using Matlab ® (Mathworks), version 7.13.

Over the entire field of view, 40 profiles perpendicular to the sources were considered for each image, with a length of 40 mm and centered around the maximum intensity pixel (Fig. 1, left). The width of the profiles was 27 pixels (30 mm) and each profile was summed in the direction of the sources.

For each profile, the maximum was determined using parabolic fit and the FWHM was determined via linear interpolation.

Statistical analysis of FWHM values was performed using R software [10].

### Measuring planar spatial resolution with tilted linear sources for 4 angles with scatter

The phantom described in the previous paragraph was used with only fillable extension tubes for linear sources.

The slotted slab was tilted by moving one corner of the slab 10, 20, 30 and 40 mm from its aligned position. These shifts correspond to tilt angles of 1.9°, 3.8°, 5.7° and 7.7° relative to the aligned position. An example of a 5.7° tilt of the sources is shown on Fig. 1 right. Sources activity and imaging parameters were identical to the NEMA acquisitions.

Additional acquisitions were performed without PMMA over the sources and with one 5-cm PMMA block over the sources to visually assess the camera’s response to tilted line sources with 4 tilt angles at distances of less than 10 cm as defined in the NEMA protocol.

As the sources are not perpendicular to the image matrix, the NEMA processing method for FWHM calculation could not be readily applied to tilted sources.

FWHM was calculated using Matlab software: the acquired images were rotated using bicubic interpolation so that the linear source images were aligned with the image axes. Profiles were summed over a length of 100 mm, chosen to be compatible with the 120 mm capillaries recommended in the NEMA protocol. The processing method for the FWHM calculation is the same as for the NEMA method, except for an additional preliminary stage to remove background noise. A Gaussian fit with an offset was applied to each profile and the background value was estimated as the offset value of this fit. This fit was not used to estimate the spatial resolution FWHM but only for the background estimation.

On each linear portion of the source, 2 FWHM values averaged on a 100 mm length were calculated, making available 10 FWHM values for each image of the phantom, as represented on Fig. 1, right.

### Characterizing spatial resolution at distances of 0 to 20 cm with tilted sources in scatter conditions

To investigate the effect of distance and scatter on spatial resolution, FWHM was measured with tilted sources at a single tilt angle of 5.7°. The slab containing the sources was moved to distances of 0, 1, 2, 3, 4, 5, 10, 15 and 20 cm from the surface of the phantom, using 1 cm and 5 cm PMMA slabs. The total thickness of the PMMA phantom was 21 cm for all measurements.

As spatial resolution was evaluated at larger distances with higher expected FWHM, the linear sources were spaced differently from the previous configuration with 4 tilts. For these measurements, only 4 linear sources were used, with intervals between the four linear sources of 60, 55 and 70 mm. The FWHM calculation was processed as previously described, except that the length of the profiles was 60 mm.

To complete the characterization of spatial resolution, images were processed with and without Clarity 2D post-processing with the standard 40% weighting factor as recommended by the manufacturer.

## Results

### Visual assessment of the camera’s response to tilted lines at different distances

**Fig. 2:**
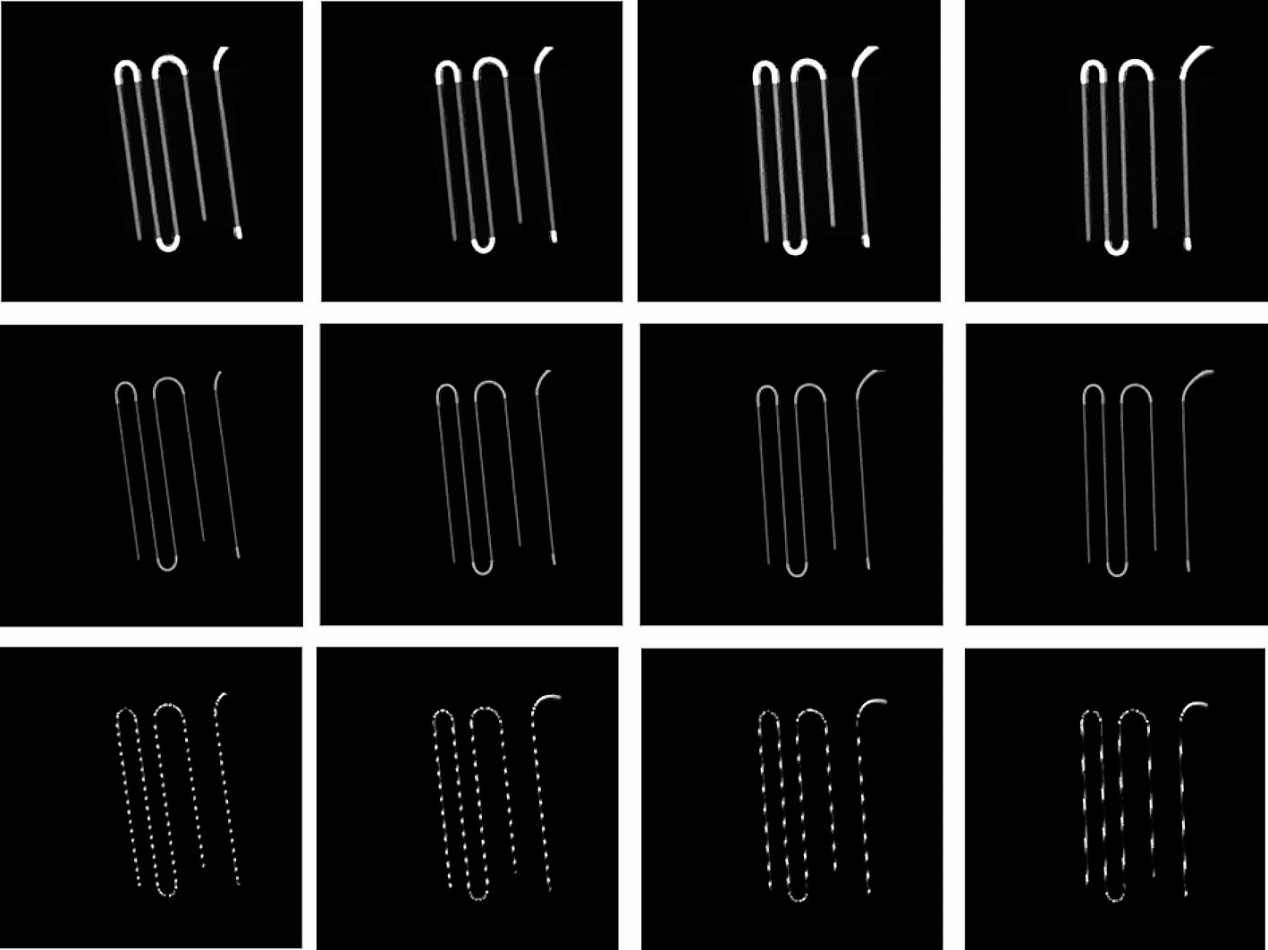
Images of sources with 4 tilt angles acquired at 3 distances, without Clarity post-processing. Sources are located at the surface (upper line), under 5 cm (middle line) and 10 cm (bottom line) of PMMA with tilts of 10 mm (1st column), 20 mm (2nd column), 30 mm (3rd column) and 40 mm (4th column) compared to its aligned position

Artifacts are visible on images with tilted sources placed on the surface of the phantom (Fig. 2, upper line): sources appear as dotted lines in the images, depending on the tilt angle of the sources. This effect is no longer visible when the sources are under 5 cm and 10 cm PMMA thicknesses (Fig. 2, middle and bottom lines).

### Comparison of spatial resolution calculated with NEMA and tilted sources

The mean FWHM estimated using the NEMA method at 10 cm was 7.63 mm. This value is very close to the FWHM specified on the manufacturer datasheet of 7.6 mm and the FWHM measured by Ito et al. of 7.8 mm in conditions close to NEMA [2]. The minimum and maximum estimated FWHMs using the NEMA method, 7.36 mm and 7.91 mm respectively, are also within the 4% tolerance specified by the manufacturer.

The mean FWHM at 10 cm (min, max) evaluated on tilted sources for the 4 tilt angles from 1.9° to 7.7° and their respective deviations from the mean NEMA FWHM are given in Table 1.

**Table 1:** FWHM estimated with 4 tilt angles and relative deviation from the mean NEMA FWHM

Tilt angle of the sources used to estimate the FWHM	Average FWHM in mm (min, max)	Average relative deviation (min, max) from the mean NEMA FWHM
1.9°	7.57 (7.20–7.94)	-0.79% (-5.64%, 4.06%)
3.8°	7.61 (7.27, 8.00)	-0.26% (-4.72%, 4.85%)
5.7°	7.61 (7.28, 7.98)	-0.26% (-4.59%, 4.59%)
7.7°	7.60 (7.38, 7.91)	-0.39% (-3.28%, 3.67%)

For each of the angles considered, the FWHM estimates are not significantly different from those obtained by the NEMA method (*p* > 0.05, Wilcoxon test).

### Variation in spatial resolution according to distance, with and without clarity processing

**Fig. 3:**
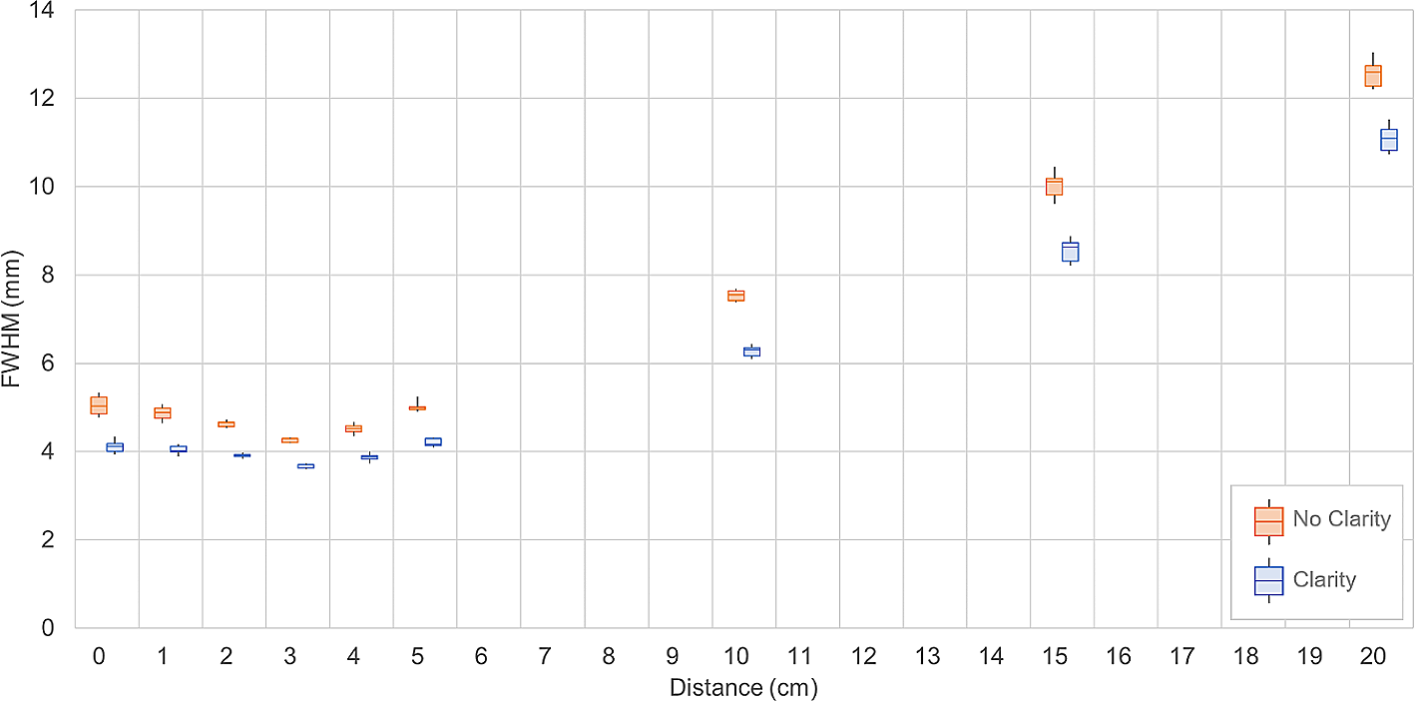
Variation in FWHM with distance, with and without Clarity post-processing. FWHM is evaluated at distances ranging from 0 to 20 cm from the collimator

To characterize the spatial resolution over a larger range of distances, FWHM values are displayed with boxplots in Fig. 3 with and without Clarity processing.

**Fig. 4:**
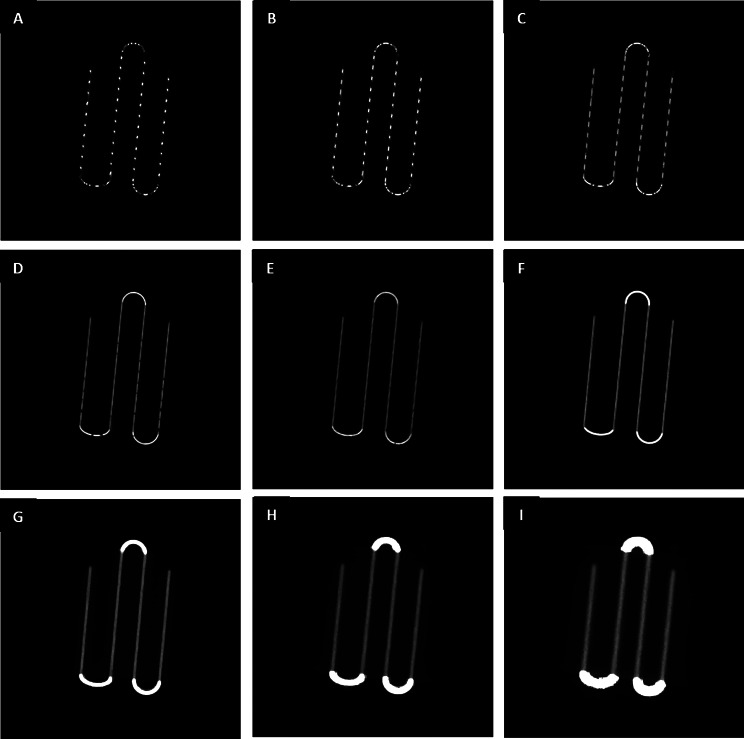
Images of tilted sources with a single angle acquired at 9 distances. Tilted linear sources are placed under 0 (**A**), 1 (**B**), 2 (**C**), 3 (**D**), 4 (**E**), 5 (**F**), 10 (**G**), 15 (**H**) and 20 (**I**) cm PMMA thickness with an angle of 5.7° and acquired without Clarity post-processing. Due to the attenuation of PMMA blocks, the portion of the extension tube filled with Tc-99 m being outside the PMMA blocks appears more intense as the source-to-detector distance increases

These results demonstrate a decrease in spatial resolution with distance in scattering conditions up to a distance of around 3 cm. This decrease is linear and similar to the distance dependency of resolution observed in scintillation cameras. For distances of less than 3 cm, the spatial resolution no longer decreases with the distance. Visually, images acquired with distances of less than 3 cm exhibit sampling artefacts (Fig. 4), with or without Clarity post-processing.

As expected, the FWHM is lower for images processed with Clarity, with an average decrease of 15.36% (min 11.70%, max 18.30%) of the measured FWHM compared to the non-processed images. The corresponding absolute difference in FWHM values between images with and without Clarity processing ranges from 0.65 (4 cm distance) to 1.48 mm (20 cm distance).

## Discussion

### Proposed measurement method

The present study focuses on the measurement and characterization of planar system spatial resolution under “user conditions” (practical, with scatter, quick to implement, at different distances) for pixelated CZT detectors. The measurement method proposed is applied using matched square WEHR collimator holes and from distances of 0 to 20 cm.

The spatial resolution evaluated with the NEMA method is a measurement at a distance of 10 cm for acceptance testing and is unsuitable for measuring spatial resolution in clinical mode across multiple distances. Alignment of sources with the detector axis is crucial when employing this method to measure spatial resolution and requires meticulous attention. Despite the presence of tracking systems on the camera (such as lines on the collimator or screens in the room), the practical alignment of sources remains cumbersome. Additionally, in user mode, the system lacks software tools to assess this alignment. Once sources are aligned, the process of shifting them by ten 1-mm increments before each acquisition is time-consuming and laborious.

### Tilted sources method in practice

We recommend using a tilt angle of 5.7° relative to the detector axes, which corresponds to applying a 1 cm offset to an aligned source of 10 cm in length. The results show that the method is not very sensitive to the angle used, within the range of angles studied. Meticulous alignment of the source lines with the detector pixels before tilting is not required.

The method’s validity was assessed through profiles drawn on a 100 mm active length, indicating its applicability with NEMA-recommended glass capillaries of 120 mm in length, for instance. A single acquisition is required for each distance to compute spatial resolution.

While we developed this method using Matlab but, alternatively, free image processing software such as Fiji can also be used [11].

### Validation with the NEMA method

The average FWHM value calculated with the proposed method is close to the average NEMA FWHM value. The estimated minimum and maximum values are not within the tolerance of +/- 4% announced by the manufacturer for the NEMA method at 10 cm.

The largest range of FWHM values observed at 10 cm (30 mm tilt: 5.7°) corresponds to deviations of +/- 4.6% from the average FWHM value. This higher amplitude is explained by a lower amount of signal used to estimate the FWHM: instead of using 10 images of a 30 mm profile for the NEMA method, a single tilted image of a 100 mm profile is used. The dispersion of values can be reduced by using a longer profile or multiple sources.

### Limits of the study

**Fig. 5:**
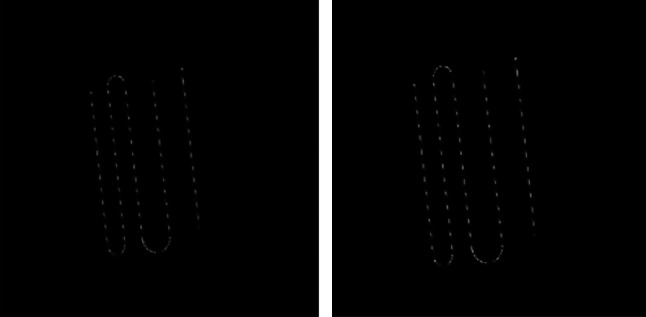
Influence of the matrix size on images of tilted sources. Acquisitions were performed with 256 × 256 (left) and 512 × 512 matrix (right)

We determined the FWHM using interpolated 512 × 512 images, as recommended by NEMA, rather than relying on the native spatial sampling of 256 × 256 from the CZT camera. However, the limitation of this approach lies in the additional information introduced by the default interpolation method using the Lanczos kernel. We did not explore the impact of the interpolation method’s parameters, but we previously verified that interpolation did not increase artifacts. For instance, Fig. 5 depicts the same test object captured under 1 cm of PMMA with native pixels (256 × 256 pixels, left) and interpolated pixels (512 × 512 matrix, right).

### Origin of artefacts seen at short distances

When imaging tilted line sources with this detector, periodic image artifacts are visible at small distances. The shape and spatial frequency of these artifacts depend on the relative position of the line source with respect to the collimator-detector system. Such artefacts have been extensively described on scintillation cameras and explained by the Classical Hole-Pattern Criterion: the hole pattern of a collimator is visible if the distance between the centers of two adjacent holes is larger than both the intrinsic resolution and the object being imaged [12]. For scintillation cameras, a localization system takes into account several adjacent detection items and the intrinsic resolution acts as a blurring effect and masks collimator holes. With CZT cameras, localizing a detected event only depends on the detector element where the photon deposits the majority of its energy. The resolution is determined by the pixel size and no longer acts as a blurring effect [13]. As a result, when the line source is directly above the open part of the collimator hole in front of a detector element, the signal is maximal, whereas a small lateral source shift results in a lower signal on that detector element.

This effect, combined with the spatial sampling of 2.46 × 2.46 mm² pixels close to the expected spatial resolution, strongly limits this detector’s characterization of spatial resolution at small distances. Under these conditions, and for this collimator, the use of line sources does not allow proper assessment of spatial resolution. Beyond a distance of around 3 cm, the artifacts are no longer visible and the spatial resolution depends linearly on the distance, as on a scintillation camera.

This effect also affects the values found regardless of the analysis method used. Using the Clarity post- processing method with the 40% default value improves this spatial resolution by 15.36% on average but does not solve this problem at close distances, related to the detector characteristics.

**Fig. 6:**
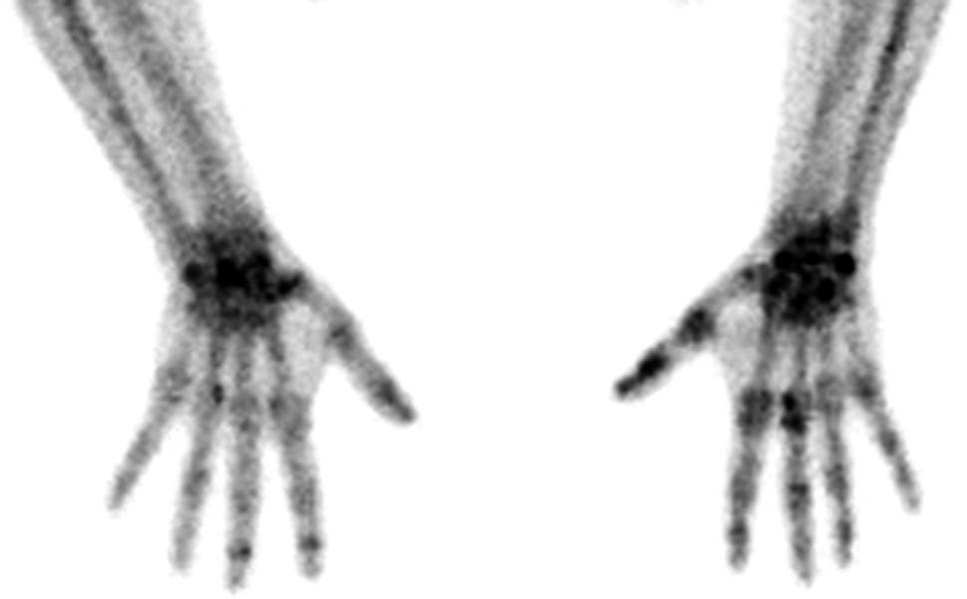
Planar bone scintigraphy of the hands acquired on the GEHC NM/CT 670 CZT camera

According to clinical experience at our centers, these artefacts are not visible on clinical images, even acquired at a very short distance to the detector. For example, in bone scintigraphy of the extremities, hands are placed directly on the detector (Fig. 6). The distribution of activity in normal bone is very different from an infinite contrast line source. One can hypothesize that the activity in and around the bones exposes the detector more homogeneously than the linear sources used in this study and the conditions of Classical Hole-Pattern Criterion are no longer met.

## Conclusion

We evaluated a practical method for assessing spatial resolution FWHM in scatter conditions adapted to a CZT gamma camera equipped with planar detectors. This method relies on the use of tilted linear sources and is simpler and faster to implement. It uses the same linear sources and gives results which are comparable to the NEMA reference method at 10 cm.

The tilted sources method was used to characterize the spatial resolution with the distance for the CZT camera equipped with a WEHR collimator and measure the influence of post-processing image-enhancement on spatial resolution.

For both methods, and in these conditions, assessing spatial resolution with linear sources at a short distance (< 3 cm) is limited by the solid angle of the collimator and by signal digitization.

## Data Availability

The datasets used and/or analyzed during the current study are available from the corresponding author on reasonable request.
